# Crystal structure of (–)-methyl (*R*,*E*)-4-[(2*R*,4*R*)-2-amino-2-tri­chloro­methyl-1,3-dioxolan-4-yl]-4-hy­droxy-2-methyl­but-2-enoate

**DOI:** 10.1107/S2056989017008283

**Published:** 2017-06-07

**Authors:** Takeshi Oishi, Mayu Kidena, Tomoya Sugai, Takaaki Sato, Noritaka Chida

**Affiliations:** a School of Medicine, Keio University, Hiyoshi 4-1-1, Kohoku-ku, Yokohama 223-8521, Japan; b Department of Applied Chemistry, Faculty of Science and Technology, Keio University, Hiyoshi 3-14-1, Kohoku-ku, Yokohama 223-8522, Japan

**Keywords:** crystal structure, 1,3-dioxolane, hy­droxy group, amino group, hydrogen bonding

## Abstract

In the title compound, C_10_H_14_Cl_3_NO_5_, the five-membered dioxolane ring adopts an envelope conformation with the C atom bonded to the butenoate side chain as the flap. It deviates from the mean plane of the other atoms in the ring by 0.446 (6) Å. In the crystal, mol­ecules are connected by O—H⋯O hydrogen bonds into helical chains running along the *b-*axis direction. The chains are linked into a sheet structure parallel to (001) by an N—H⋯O hydrogen bond. These classical hydrogen bonds enclose an *R* ^4^_4_(24) graph-set motif in the sheet structure. Furthermore, a weak inter­molecular C—H⋯Cl inter­action expands the sheet structures into a three-dimensional network.

## Abstract 2

In the title compound, the 1,3-dioxane ring has an envelope conformation. In the crystal, classical O—H⋯O and N—H⋯O hydrogen bonds link mol­ecules into a sheet structure, and a weak inter­molecular C—H⋯Cl inter­action extends the sheet structure into a three-dimensional network.

## Chemical context   

Cyclic compounds often play a significant role, not only in controlling stereochemistry due to their conformational rigidity, but also as protecting groups in organic synthesis. On the basis of this concept, we have explored the utilization of cyclic ortho­amides, prepared from allylic diol and triol with known conditions (Overman, 1974 ▸; 1976 ▸), and have developed a new strategy for the total synthesis of a certain natural product (Nakayama, *et al.*, 2013 ▸). The title compound is a structural isomer of a recently reported compound (Oishi *et al.*, 2016 ▸).
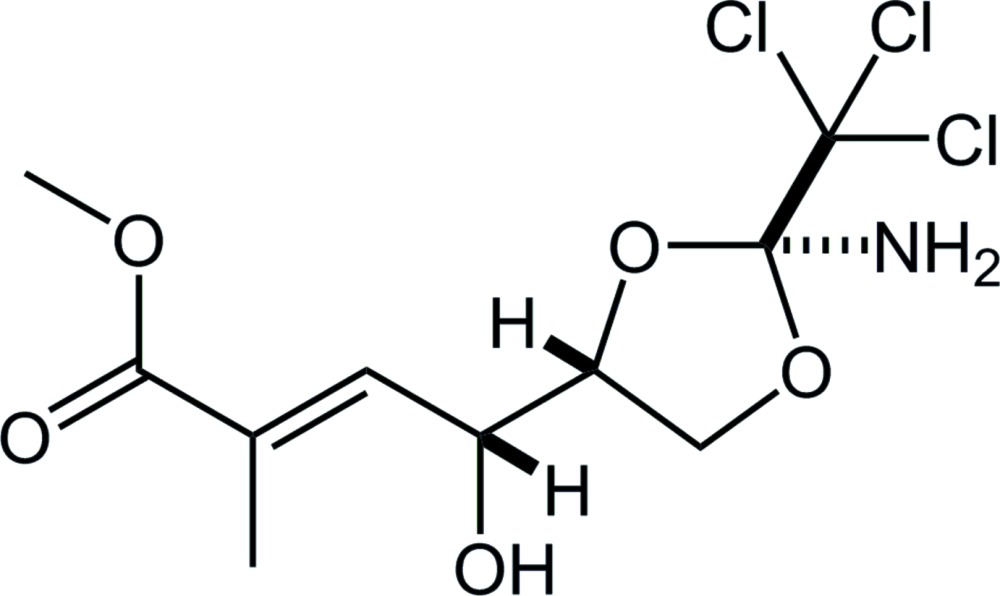


## Structural commentary   

**Figure 1:**
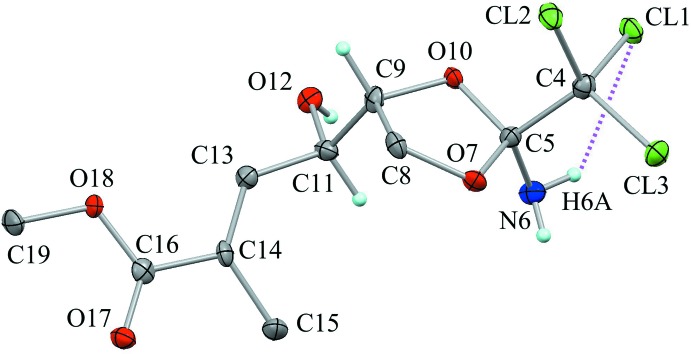
The mol­ecular structure of the title compound, with the atom labelling. Displacement ellipsoids are drawn at the 50% probability level. The purple dotted line indicates the short intra­molecular N—H⋯Cl contact (see Table 1 ▸). Only H atoms connected to N, O and chiral C atoms are shown for clarity.

The mol­ecular structure of the title compound is shown in Fig. 1 ▸. The 1,3-dioxolane ring (C5/O7/C8/C9/O10) adopts an envelope conformation with the flap atom C9 deviating by 0.446 (6) Å from the mean plane of the other four atoms [puckering parameters are *Q*(2) = 0.285 (4) Å and *φ*(2) = 296.7 (8)°]. The C=C and C=O double bonds of the unsat­urated ester are slightly skewed with torsion angle C13=C14—C16=O18 being of 8.4 (6)°. There is a weak intra­molecular N6—H6*A*⋯Cl1 inter­action present (Table 1 ▸).

## Supra­molecular features   

**Figure 2:**
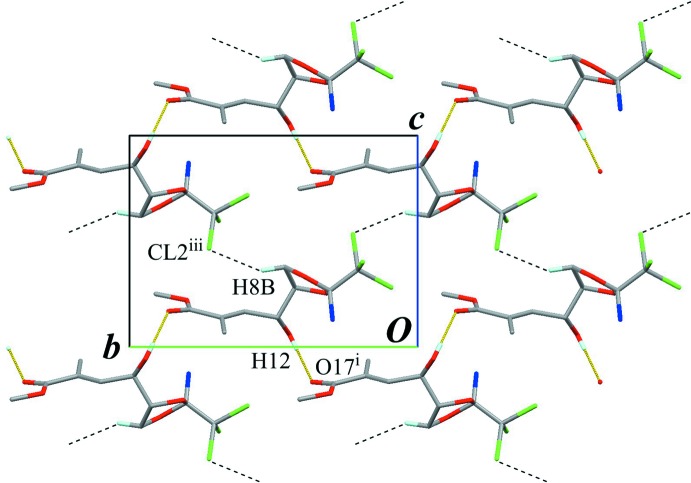
The crystal packing of the title compound, viewed along the *a* axis, showing the helical chain structures running along the *b-*axis direction. Yellow lines indicate the inter­molecular O—H⋯O hydrogen bonds. Black dashed lines indicate weak inter­molecular C—H⋯Cl inter­actions. Only H atoms involved in the hydrogen bonds are shown for clarity. [Symmetry codes: (i) −*x* + 1, *y* – 1/2, −*z*; (iii) −*x* + 1, *y* + 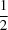, −*z* + 1.]

**Figure 3:**
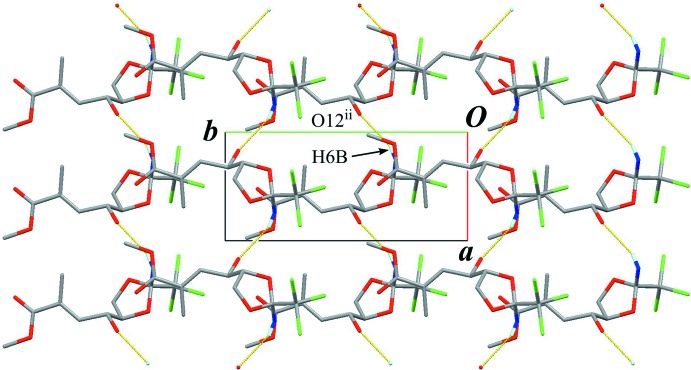
The crystal packing of the title compound, viewed along the *c* axis, showing the sheet structure parallel to (001). The helical chain running along the *b-*axis direction is drawn as overlapped mol­ecules. Yellow lines indicate the inter­molecular N—H⋯O hydrogen bonds. Only H atoms involved in the hydrogen bonds are shown for clarity. [Symmetry code: (ii) *x* – 1, *y*, *z*.]

**Figure 4:**
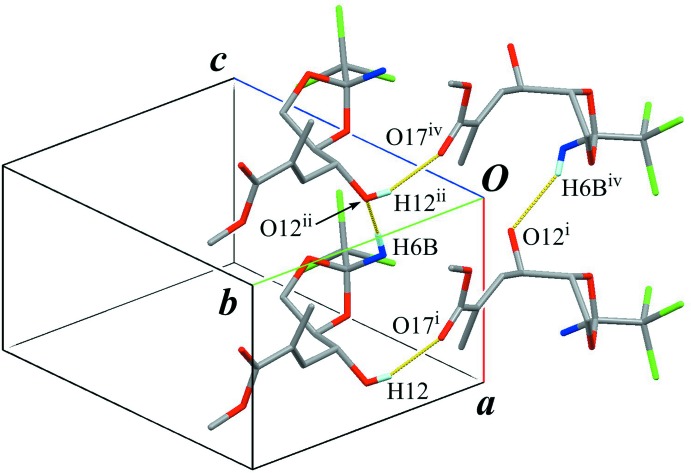
A part of sheet structure, showing the 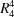 graph-set motif generated by classical O—H⋯O and N—H⋯O hydrogen bonds. Yellow lines indicate the inter­molecular O—H⋯O and N—H⋯O hydrogen bonds. Only H atoms involved in the hydrogen bonds are shown for clarity. [Symmetry codes: (i) −*x* + 1, *y* – 1/2, −*z*; (ii) *x* – 1, *y*, *z*; (iv) −*x*, *y* − 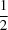, −*z*.]

In the crystal, a classical O—H⋯O hydrogen bond (O12—H12⋯O17^i^; Table 1 ▸) connects the mol­ecules into a helical-chain running along the *b-*axis direction, with a *C*(7) graph-set motif (Fig. 2 ▸). A classical N—H⋯O hydrogen bond (N6—H6*B*⋯O12^ii^; Table 1 ▸), which is formed between one of N-bound H atoms and hy­droxy O group, links the chains into a sheet structure parallel to (001), also generating a *C*(7) graph-set motif (Fig. 3 ▸). In the sheet structure, the classical O—H⋯O and N—H⋯O hydrogen bonds enclose an 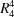
(24) graph-set motif (Fig. 4 ▸). Furthermore, a weak C—H⋯Cl inter­action (C8—H8*B*⋯Cl2^iii^; Table 1 ▸) supports the crystal packing to construct a three-dimensional architecture (Fig. 2 ▸). An inter­molecular Cl1⋯O17 (*x*, *y* − 1, *z*) short contact of 3.076 (3) Å is also observed.

## Database survey   

**Figure 5:**
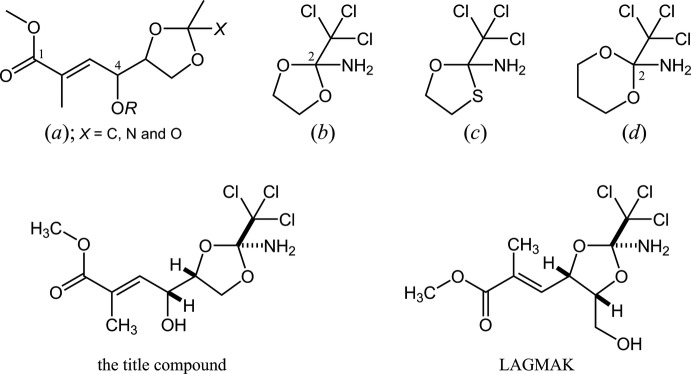
The core structures for the database survey; (*a*) 4-alk­oxy-2-methyl-4-(2-methyl-1,3-dioxolan-4-yl)but-2-enoate, (*b*) 2-amino-2-tri­chloro­methyl-1,3-dioxolane, and its (*c*) -1,3-oxa­thiol­ane and (*d*) -1,3-dioxane derivatives instead of the 1,3-dioxolane ring.

In the Cambridge Structural Database (CSD, Version 5.38, Feb. 2017; Groom *et al.*, 2016 ▸), there are two structures containing the 4-alk­oxy-2-methyl-4-(2-methyl-1,3-dioxolan-4-yl)but-2-enoate skeleton, (*a*), related to the title compound (Fig. 5 ▸), but its 4-hy­droxy free derivative (*R* = H) has not yet been reported.

**Figure 6:**
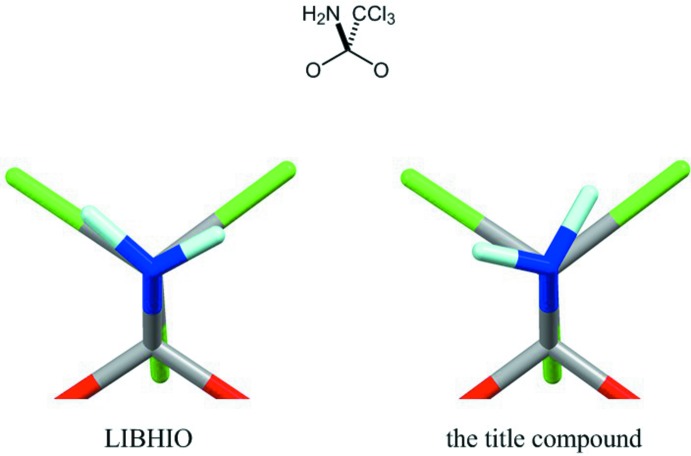
A projected diagram looking through the N atom of the amino group onto the C atom of the tri­chloro­methyl group.

For the cyclic ortho­amide core with a tri­chloro­methyl group on the central carbon atom, four structures are registered in the CSD. These are two derivatives (WEKWOY: Haeckel *et al.*, 1994 ▸; and LAGMAK: Oishi *et al.*, 2016 ▸) of 1,3-dioxolane (*b*), one derivative (WAXBEE: Metwally, 2011 ▸) of 1,3-oxa­thiol­ane (*c*), and one derivative (LIBHIO: Rondot *et al.*, 2007 ▸) of 1,3-dioxane (*d*). The amino H atoms were refined as adopting an *sp* ^2^ configuration for WEKWOY and WAXBEE, while they were refined assuming an *sp* ^3^ configuration of the N atom for LIBHIO and LAGMAK, as in the present study. Each N—H bond of the amino group in LIBHIO is mostly eclipsed by the neighbouring C—Cl bonds of the tri­chloro­methyl group, whereas those in the title compound are slightly tilted (Fig. 6 ▸). There is an intra­molecular N—H⋯Cl inter­action [H6*A*⋯Cl1 = 2.66 (4) Å; N6—H6*A*⋯Cl1 = 115 (3)°] in the title compound (Table 1 ▸), while the corres­ponding geometries are 2.76 Å and 109° in LIBHIO. These amino groups may be oriented to avoid intra­molecular non-bonding short contacts as well as to form classical inter­molecular hydrogen bonds. The amino H atoms in LAGMAK are disordered according to the possible intra­molecular N—H⋯O and N⋯H—O hydrogen bonds with the hy­droxy group (Oishi *et al.*, 2016 ▸).

## Synthesis and crystallization   

The title compound was afforded from l-threose, which can be prepared according to the reported procedure (Smith *et al.*, 1992 ▸) from d-galactose (Kidena *et al.*, 2017 ▸). Purification was carried out by silica gel column chromatography, and colourless crystals were obtained from a benzene solution under a hexane-saturated atmosphere, by slow evaporation at ambient temperature (m.p. 358–359 K). [*α*]_D_^24^ – 32.7 (*c* 1.01, CHCl_3_). HRMS (ESI) *m*/*z* calculated for C_10_H_15_Cl_3_NO_5_^+^ [*M* + H]^+^: 334.0016; found: 334.0016.

## Refinement   

Crystal data, data collection and structure refinement details are summarized in Table 2 ▸. C-bound H atoms were positioned geometrically with C—H = 0.95–1.00 Å, and constrained to ride on their parent atoms with *U*_iso_(H) = 1.5*U*_eq_(methyl C) and 1.2*U*_eq_(C) for other C-bound H atoms. The hy­droxy H atom was placed, guided by difference-Fourier maps, with O—H = 0.84 Å and refined with *U*_iso_(H) = 1.5*U*_eq_(O). The amino H atoms were placed, guided by difference-Fourier maps, and were refined with distance restraints of N—H = 0.86 (2) Å and H⋯H = 1.40 (2) Å, with *U*_iso_(H) = 1.2*U*_eq_(N).

## Supplementary Material

Crystal structure: contains datablock(s) global, I. DOI: 10.1107/S2056989017008283/su5377sup1.cif

Structure factors: contains datablock(s) I. DOI: 10.1107/S2056989017008283/su5377Isup2.hkl

Supporting information file. DOI: 10.1107/S2056989017008283/su5377Isup3.cml

CCDC reference: 1554119

Additional supporting information:  crystallographic information; 3D view; checkCIF report

## Figures and Tables

**Table 1:** Hydrogen-bond geometry (Å, °)

*D*—H⋯*A*	*D*—H	H⋯*A*	*D*⋯*A*	*D*—H⋯*A*
N6—H6*A*⋯Cl1	0.87 (2)	2.66 (4)	3.118 (4)	115 (3)
O12—H12⋯O17^i^	0.84	1.97	2.774 (4)	161
N6—H6*B*⋯O12^ii^	0.84 (2)	2.28 (3)	3.047 (5)	152 (4)
C8—H8*B*⋯Cl2^iii^	0.99	2.83	3.713 (5)	149

Symmetry codes: (i) 
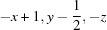
; (ii) 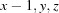; (iii) 
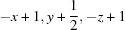
.

**Table 2:** Experimental details

Crystal data	
Chemical formula	C_10_H_14_Cl_3_NO_5_
*M*_r_	334.57
Crystal system, space group	Monoclinic, *P*2_1_
Temperature (K)	90
*a*, *b*, *c* (Å)	5.8494 (4), 12.6458 (8), 9.5658 (6)
β (°)	104.763 (2)
*V* (Å^3^)	684.23 (8)
*Z*	2
Radiation type	Mo *K*α
μ (mm^−1^)	0.68
Crystal size (mm)	0.28 × 0.22 × 0.08

Data collection	
Diffractometer	Bruker D8 Venture
Absorption correction	Multi-scan (*SADABS*; Bruker, 2016 ▸)
*T*_min_, *T*_max_	0.83, 0.95
No. of measured, independent and observed [*I* > 2σ(*I*)] reflections	10686, 2376, 2268
*R*_int_	0.042
(sin θ/λ)_max_ (Å^−1^)	0.595

Refinement	
*R*[*F* ^2^ > 2σ(*F* ^2^)], *wR*(*F* ^2^), *S*	0.030, 0.060, 1.06
No. of reflections	2376
No. of parameters	181
No. of restraints	4
H-atom treatment	H atoms treated by a mixture of independent and constrained refinement
Δρ_max_, Δρ_min_ (e Å^−3^)	0.28, −0.29
Absolute structure	Flack *x* determined using 993 quotients [(*I* ^+^)−(*I* ^−^)]/[(*I* ^+^)+(*I* ^−^)] (Parsons *et al.*, 2013 ▸).
Absolute structure parameter	0.04 (3)

Computer programs: *APEX3* and *SAINT* (Bruker, 2016 ▸), *SHELXT2014/5* (Sheldrick, 2015*a* ▸), *SHELXL2014/7* (Sheldrick, 2015*b* ▸), *Mercury* (Macrae *et al.*, 2008 ▸), *publCIF* (Westrip, 2010 ▸) and *PLATON* (Spek, 2009 ▸).

## supplementary crystallographic information

### Crystal data

**Table:**

C_10_H_14_Cl_3_NO_5_	*D*_x_ = 1.624 Mg m^−^^3^
*M**_r_* = 334.57	Melting point = 358–359 K
Monoclinic, *P*2_1_	Mo *K*α radiation, λ = 0.71073 Å
*a* = 5.8494 (4) Å	Cell parameters from 8568 reflections
*b* = 12.6458 (8) Å	θ = 2.7–25.4°
*c* = 9.5658 (6) Å	µ = 0.68 mm^−^^1^
β = 104.763 (2)°	*T* = 90 K
*V* = 684.23 (8) Å^3^	Plate, colorless
*Z* = 2	0.28 × 0.22 × 0.08 mm
*F*(000) = 344	

### Data collection

**Table:**

Bruker D8 Venture diffractometer	2376 independent reflections
Radiation source: fine-focus sealed tube	2268 reflections with *I* > 2σ(*I*)
Multilayered confocal mirror monochromator	*R*_int_ = 0.042
Detector resolution: 7.4074 pixels mm^-1^	θ_max_ = 25.0°, θ_min_ = 2.2°
φ and ω scans	*h* = −6→6
Absorption correction: multi-scan (*SADABS*; Bruker, 2016)	*k* = −15→15
*T*_min_ = 0.83, *T*_max_ = 0.95	*l* = −11→11
10686 measured reflections	

### Refinement

**Table:**

Refinement on *F*^2^	Secondary atom site location: difference Fourier map
Least-squares matrix: full	Hydrogen site location: mixed
*R*[*F*^2^ > 2σ(*F*^2^)] = 0.030	H atoms treated by a mixture of independent and constrained refinement
*wR*(*F*^2^) = 0.060	*w* = 1/[σ^2^(*F*_o_^2^) + 0.7994*P*] where *P* = (*F*_o_^2^ + 2*F*_c_^2^)/3
*S* = 1.06	(Δ/σ)_max_ < 0.001
2376 reflections	Δρ_max_ = 0.28 e Å^−^^3^
181 parameters	Δρ_min_ = −0.29 e Å^−^^3^
4 restraints	Absolute structure: Flack *x* determined using 993 quotients [(*I*^+^)-(*I*^-^)]/[(*I*^+^)+(*I*^-^)] (Parsons *et al.*, 2013).
Primary atom site location: structure-invariant direct methods	Absolute structure parameter: 0.04 (3)

### Special details

**Table:**

Experimental. IR (film): 3393, 3325, 2953, 1714, 1438, 1239, 1093, 1035, 825, 803, 749 cm^-1^; ^1^H NMR (500 MHz, CDCl_3_): *δ* (p.p.m.) 6.78 (dq, *J* = 8.7, 1.4 Hz, 1H; H13), 4.69–4.62 (m, 1H; H12), 4.65 (ddd, *J* = 7.0, 4.3, 2.9 Hz, 1H; H9), 4.43–4.39 (m, 3H; H8AB & H11), 3.75 (s, 3H; H19ABC), 2.95 (bs, 2H; H6AB), 1.92 (d, *J* = 1.4 Hz, 3H; H14ABC); ^13^C NMR (125 MHz, CDCl_3_): *δ* (p.p.m.) 168.2 (C; C16), 138.9 (CH; C13), 130.2 (C; C14), 116.0 (C; C5), 103.3 (C; C4), 82.9 (CH; C9), 70.1 (CH_2_; C8), 69.2 (CH; C11), 52.2 (CH_3_; C19), 13.2 (CH_3_; C14).
Geometry. All esds (except the esd in the dihedral angle between two l.s. planes) are estimated using the full covariance matrix. The cell esds are taken into account individually in the estimation of esds in distances, angles and torsion angles; correlations between esds in cell parameters are only used when they are defined by crystal symmetry. An approximate (isotropic) treatment of cell esds is used for estimating esds involving l.s. planes.
Refinement. Refinement of *F*^2^ against ALL reflections. The weighted *R*-factor *wR* and goodness of fit *S* are based on *F*^2^, conventional *R*-factors *R* are based on *F*, with *F* set to zero for negative *F*^2^. The threshold expression of *F*^2^ > 2*σ*(*F*^2^) is used only for calculating *R*-factors(gt) *etc.* and is not relevant to the choice of reflections for refinement. *R*-factors based on *F*^2^ are statistically about twice as large as those based on *F*, and *R*-factors based on ALL data will be even larger.

### Fractional atomic coordinates and isotropic or equivalent isotropic displacement parameters (Å^2^)

**Table:**

	*x*	*y*	*z*	*U*_iso_*/*U*_eq_	
Cl1	0.49362 (19)	0.09132 (8)	0.28126 (12)	0.0182 (2)	
Cl2	0.63815 (19)	0.22438 (8)	0.53302 (11)	0.0188 (3)	
Cl3	0.14274 (19)	0.19083 (8)	0.40183 (12)	0.0218 (3)	
C4	0.4229 (7)	0.2071 (3)	0.3659 (4)	0.0158 (9)	
C5	0.4173 (7)	0.3050 (3)	0.2656 (5)	0.0136 (9)	
N6	0.2535 (7)	0.2954 (3)	0.1287 (4)	0.0173 (8)	
H6A	0.236 (7)	0.230 (2)	0.099 (5)	0.021*	
H6B	0.121 (6)	0.321 (3)	0.128 (5)	0.021*	
O7	0.3602 (5)	0.3953 (2)	0.3345 (3)	0.0151 (7)	
C8	0.5670 (8)	0.4608 (3)	0.3782 (5)	0.0163 (10)	
H8A	0.6482	0.4492	0.4812	0.02*	
H8B	0.5255	0.5366	0.3633	0.02*	
C9	0.7202 (8)	0.4255 (3)	0.2809 (4)	0.0149 (9)	
H9	0.8909	0.4302	0.334	0.018*	
O10	0.6524 (5)	0.3163 (2)	0.2537 (3)	0.0136 (6)	
C11	0.6763 (8)	0.4863 (3)	0.1386 (5)	0.0146 (10)	
H11	0.508	0.4774	0.0826	0.018*	
O12	0.8311 (5)	0.4449 (2)	0.0574 (3)	0.0162 (7)	
H12	0.7506	0.417	−0.019	0.024*	
C13	0.7305 (7)	0.6008 (3)	0.1666 (4)	0.0142 (9)	
H13	0.8909	0.6187	0.2091	0.017*	
C14	0.5772 (7)	0.6801 (4)	0.1381 (4)	0.0137 (9)	
C15	0.3168 (7)	0.6713 (4)	0.0683 (5)	0.0198 (10)	
H15A	0.282	0.7022	−0.0288	0.03*	
H15B	0.2282	0.7093	0.1268	0.03*	
H15C	0.2704	0.5966	0.0615	0.03*	
C16	0.6582 (8)	0.7912 (3)	0.1764 (5)	0.0146 (10)	
O17	0.5238 (5)	0.8654 (2)	0.1682 (3)	0.0165 (7)	
O18	0.8922 (5)	0.8021 (2)	0.2209 (3)	0.0151 (7)	
C19	0.9749 (8)	0.9094 (3)	0.2504 (5)	0.0209 (11)	
H19A	1.1481	0.9103	0.2763	0.031*	
H19B	0.918	0.9378	0.3307	0.031*	
H19C	0.9147	0.953	0.1641	0.031*	

### Atomic displacement parameters (Å^2^)

**Table:**

	*U*^11^	*U*^22^	*U*^33^	*U*^12^	*U*^13^	*U*^23^
Cl1	0.0221 (6)	0.0107 (5)	0.0232 (6)	0.0009 (5)	0.0080 (5)	−0.0014 (5)
Cl2	0.0220 (6)	0.0165 (6)	0.0159 (5)	−0.0020 (5)	0.0014 (4)	0.0028 (5)
Cl3	0.0160 (6)	0.0218 (6)	0.0312 (6)	−0.0003 (5)	0.0124 (5)	0.0039 (5)
C4	0.015 (2)	0.017 (3)	0.017 (2)	−0.0005 (19)	0.0063 (18)	−0.001 (2)
C5	0.012 (2)	0.012 (2)	0.016 (2)	−0.0005 (19)	0.0046 (19)	−0.0001 (19)
N6	0.0160 (19)	0.017 (2)	0.018 (2)	0.0019 (17)	0.0027 (17)	−0.0012 (17)
O7	0.0155 (16)	0.0100 (16)	0.0208 (16)	0.0031 (13)	0.0068 (13)	−0.0037 (13)
C8	0.022 (2)	0.011 (2)	0.016 (2)	−0.002 (2)	0.0044 (19)	−0.0007 (19)
C9	0.016 (2)	0.012 (2)	0.016 (2)	−0.0047 (18)	0.0028 (18)	−0.0050 (19)
O10	0.0141 (15)	0.0077 (15)	0.0204 (16)	0.0000 (12)	0.0068 (13)	0.0006 (12)
C11	0.014 (2)	0.013 (2)	0.016 (2)	0.0026 (18)	0.0025 (19)	0.0009 (19)
O12	0.0160 (16)	0.0174 (17)	0.0154 (16)	−0.0006 (13)	0.0042 (13)	−0.0033 (14)
C13	0.014 (2)	0.014 (2)	0.015 (2)	−0.0002 (19)	0.0040 (18)	−0.002 (2)
C14	0.018 (2)	0.011 (2)	0.013 (2)	−0.003 (2)	0.0053 (18)	0.0030 (19)
C15	0.014 (2)	0.016 (2)	0.030 (3)	0.0027 (19)	0.006 (2)	0.000 (2)
C16	0.018 (2)	0.017 (2)	0.010 (2)	0.001 (2)	0.0072 (18)	0.002 (2)
O17	0.0167 (16)	0.0138 (16)	0.0186 (16)	0.0034 (14)	0.0034 (13)	0.0005 (14)
O18	0.0124 (16)	0.0105 (15)	0.0208 (16)	−0.0026 (13)	0.0016 (13)	−0.0004 (13)
C19	0.020 (3)	0.009 (2)	0.030 (3)	−0.0012 (19)	0.000 (2)	−0.003 (2)

### Geometric parameters (Å, º)

**Table:**

Cl1—C4	1.773 (4)	C11—C13	1.492 (6)
Cl2—C4	1.778 (4)	C11—H11	1.0
Cl3—C4	1.770 (4)	O12—H12	0.84
C4—C5	1.561 (6)	C13—C14	1.327 (6)
C5—O7	1.401 (5)	C13—H13	0.95
C5—O10	1.416 (5)	C14—C16	1.498 (6)
C5—N6	1.417 (6)	C14—C15	1.503 (6)
N6—H6A	0.87 (2)	C15—H15A	0.98
N6—H6B	0.84 (2)	C15—H15B	0.98
O7—C8	1.438 (5)	C15—H15C	0.98
C8—C9	1.514 (6)	C16—O17	1.213 (5)
C8—H8A	0.99	C16—O18	1.333 (5)
C8—H8B	0.99	O18—C19	1.444 (5)
C9—O10	1.442 (5)	C19—H19A	0.98
C9—C11	1.527 (6)	C19—H19B	0.98
C9—H9	1.0	C19—H19C	0.98
C11—O12	1.433 (5)		
			
C5—C4—Cl3	109.6 (3)	O12—C11—C13	108.4 (3)
C5—C4—Cl1	110.2 (3)	O12—C11—C9	108.3 (3)
Cl3—C4—Cl1	109.1 (2)	C13—C11—C9	110.4 (3)
C5—C4—Cl2	110.8 (3)	O12—C11—H11	109.9
Cl3—C4—Cl2	108.6 (2)	C13—C11—H11	109.9
Cl1—C4—Cl2	108.5 (2)	C9—C11—H11	109.9
O7—C5—O10	108.0 (3)	C11—O12—H12	109.5
O7—C5—N6	108.5 (3)	C14—C13—C11	126.5 (4)
O10—C5—N6	112.1 (3)	C14—C13—H13	116.7
O7—C5—C4	109.1 (3)	C11—C13—H13	116.7
O10—C5—C4	105.1 (3)	C13—C14—C16	120.2 (4)
N6—C5—C4	113.8 (4)	C13—C14—C15	126.2 (4)
C5—N6—H6A	112 (3)	C16—C14—C15	113.6 (4)
C5—N6—H6B	112 (3)	C14—C15—H15A	109.5
H6A—N6—H6B	109 (4)	C14—C15—H15B	109.5
C5—O7—C8	108.8 (3)	H15A—C15—H15B	109.5
O7—C8—C9	103.7 (3)	C14—C15—H15C	109.5
O7—C8—H8A	111.0	H15A—C15—H15C	109.5
C9—C8—H8A	111.0	H15B—C15—H15C	109.5
O7—C8—H8B	111.0	O17—C16—O18	122.5 (4)
C9—C8—H8B	111.0	O17—C16—C14	123.3 (4)
H8A—C8—H8B	109.0	O18—C16—C14	114.2 (4)
O10—C9—C8	102.4 (3)	C16—O18—C19	115.2 (3)
O10—C9—C11	110.2 (3)	O18—C19—H19A	109.5
C8—C9—C11	114.1 (4)	O18—C19—H19B	109.5
O10—C9—H9	110.0	H19A—C19—H19B	109.5
C8—C9—H9	110.0	O18—C19—H19C	109.5
C11—C9—H9	110.0	H19A—C19—H19C	109.5
C5—O10—C9	108.0 (3)	H19B—C19—H19C	109.5
			
Cl3—C4—C5—O7	59.6 (4)	C4—C5—O10—C9	−131.3 (3)
Cl1—C4—C5—O7	179.7 (3)	C8—C9—O10—C5	27.3 (4)
Cl2—C4—C5—O7	−60.2 (4)	C11—C9—O10—C5	−94.5 (4)
Cl3—C4—C5—O10	175.2 (3)	O10—C9—C11—O12	−65.3 (4)
Cl1—C4—C5—O10	−64.7 (3)	C8—C9—C11—O12	−179.8 (3)
Cl2—C4—C5—O10	55.5 (4)	O10—C9—C11—C13	176.1 (3)
Cl3—C4—C5—N6	−61.8 (4)	C8—C9—C11—C13	61.6 (5)
Cl1—C4—C5—N6	58.4 (4)	O12—C11—C13—C14	127.4 (4)
Cl2—C4—C5—N6	178.5 (3)	C9—C11—C13—C14	−114.1 (5)
O10—C5—O7—C8	−4.9 (4)	C11—C13—C14—C16	178.3 (4)
N6—C5—O7—C8	−126.7 (4)	C11—C13—C14—C15	−1.5 (7)
C4—C5—O7—C8	108.8 (3)	C13—C14—C16—O17	−171.5 (4)
C5—O7—C8—C9	21.5 (4)	C15—C14—C16—O17	8.3 (6)
O7—C8—C9—O10	−29.3 (4)	C13—C14—C16—O18	8.4 (6)
O7—C8—C9—C11	89.7 (4)	C15—C14—C16—O18	−171.7 (3)
O7—C5—O10—C9	−14.9 (4)	O17—C16—O18—C19	−3.5 (6)
N6—C5—O10—C9	104.6 (4)	C14—C16—O18—C19	176.6 (3)

### Hydrogen-bond geometry (Å, º)

**Table:**

*D*—H···*A*	*D*—H	H···*A*	*D*···*A*	*D*—H···*A*
N6—H6*A*···Cl1	0.87 (2)	2.66 (4)	3.118 (4)	115 (3)
O12—H12···O17^i^	0.84	1.97	2.774 (4)	161
N6—H6*B*···O12^ii^	0.84 (2)	2.28 (3)	3.047 (5)	152 (4)
C8—H8*B*···Cl2^iii^	0.99	2.83	3.713 (5)	149

Symmetry codes: (i) −*x*+1, *y*−1/2, −*z*; (ii) *x*−1, *y*, *z*; (iii) −*x*+1, *y*+1/2, −*z*+1.
